# Supplementary material for: An inducible CRISPR interference library for genetic interrogation of Saccharomyces cerevisiae biology
Source: Commun Biol. 2020 Nov 27;3:723. doi: 10.1038/s42003-020-01452-9 (PMC7695836; doi:10.1038/s42003-020-01452-9)
Supplement: Supplementary file 1 — Supplementary Information [file 42003_2020_1452_MOESM1_ESM.pdf]

An inducible CRISPR-interference library for genetic interrogation of *Saccharomyces cerevisiae* biology

Amir Momen-Roknabadi, Panos Oikonomou, Maxwell Zegans, Saeed Tavazoie

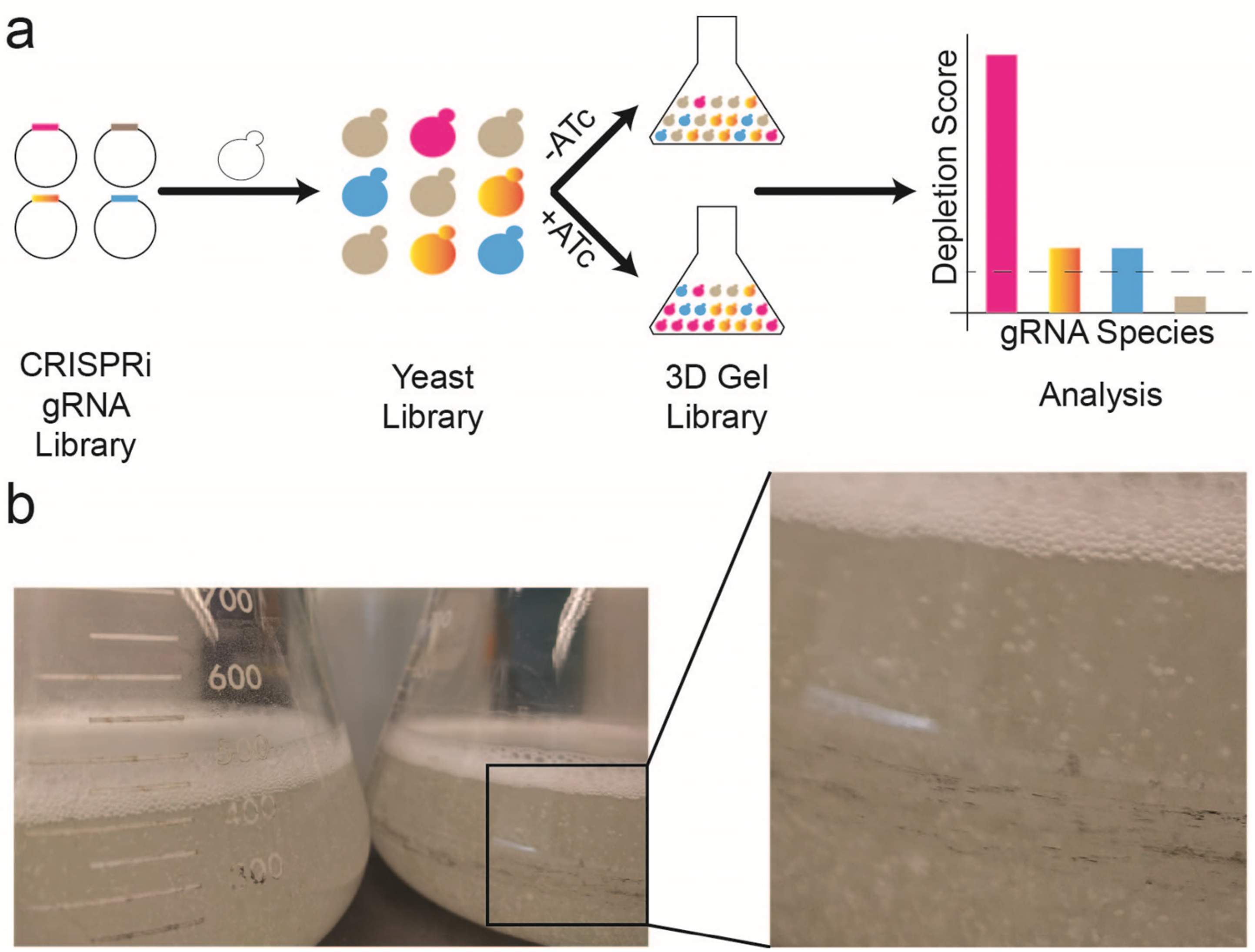

Supplementary Figure 1. Pipeline for generation and uses of whole-genome inducible CRISPRi library. a) The CRISPRi gRNA library was used to transform the yeast strain. The transformed library was grown in the semisolid 3D gel with and without ATc. The enrichment and depletion of guide sequences were profiled by next-generation sequencing. **b)** Individual yeast colonies are visible after two days growth in the semisolid media.

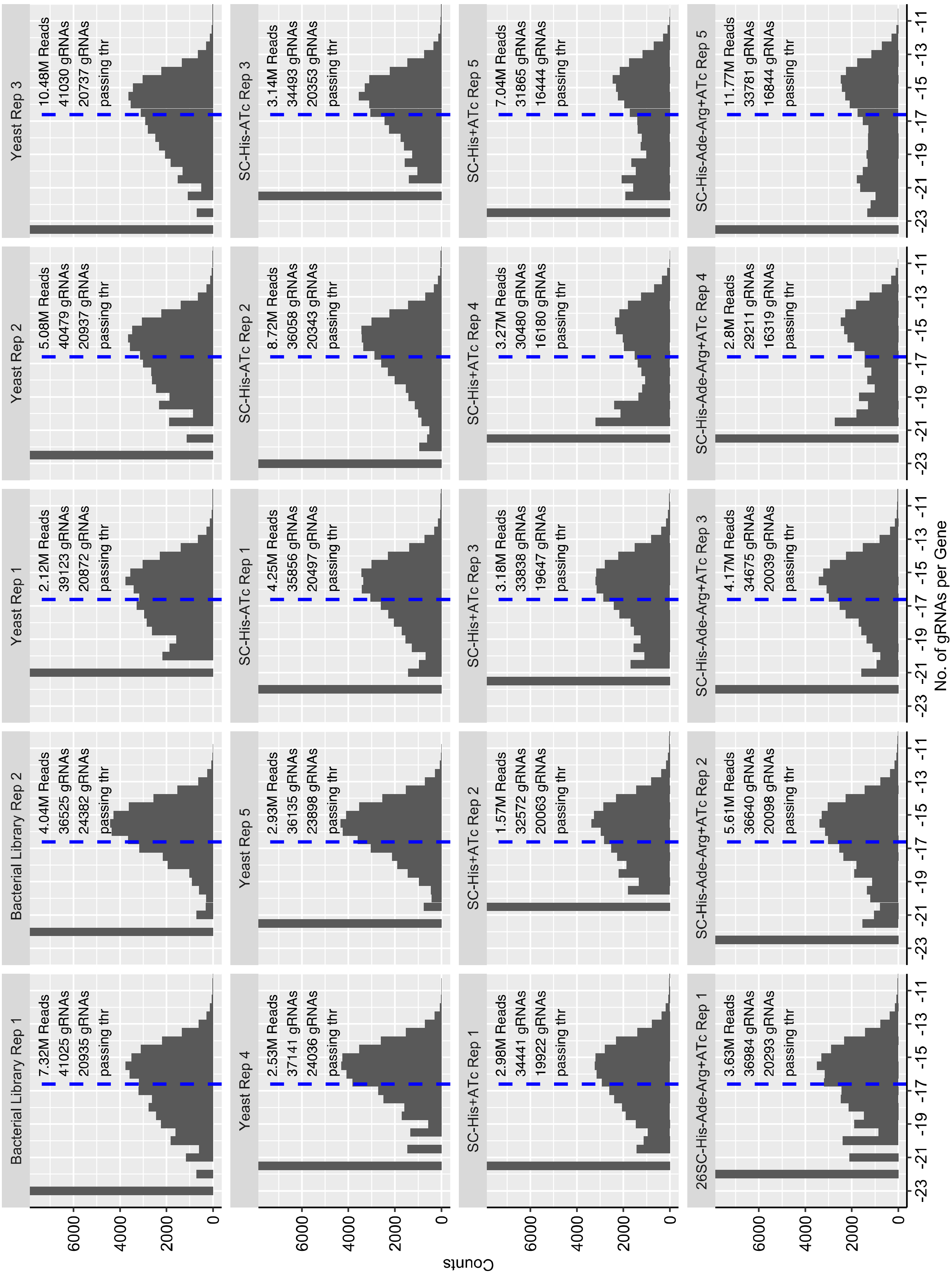

Supplementary Figure 2. Histograms depicting the frequency of gRNA read distributions in each sample. The total number of reads and gRNAs present for each sample, and the number of gRNAs with read frequency above the threshold is reported accordingly. Dashed line corresponds to the minimum read frequency threshold ~ log2(10^{-5}).

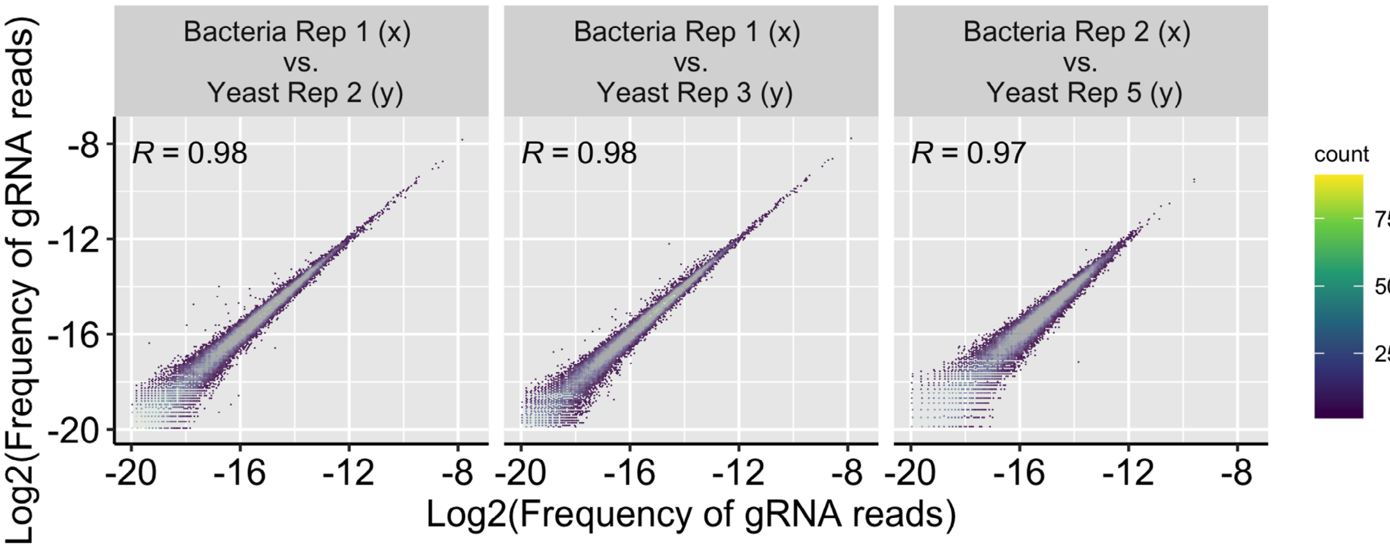

Supplementary Figure 3. Scatter plot depicting the frequency of reads per gRNA between biological replicates of the CRISPRi library. Pearson Correlation R value is reported for each pair.

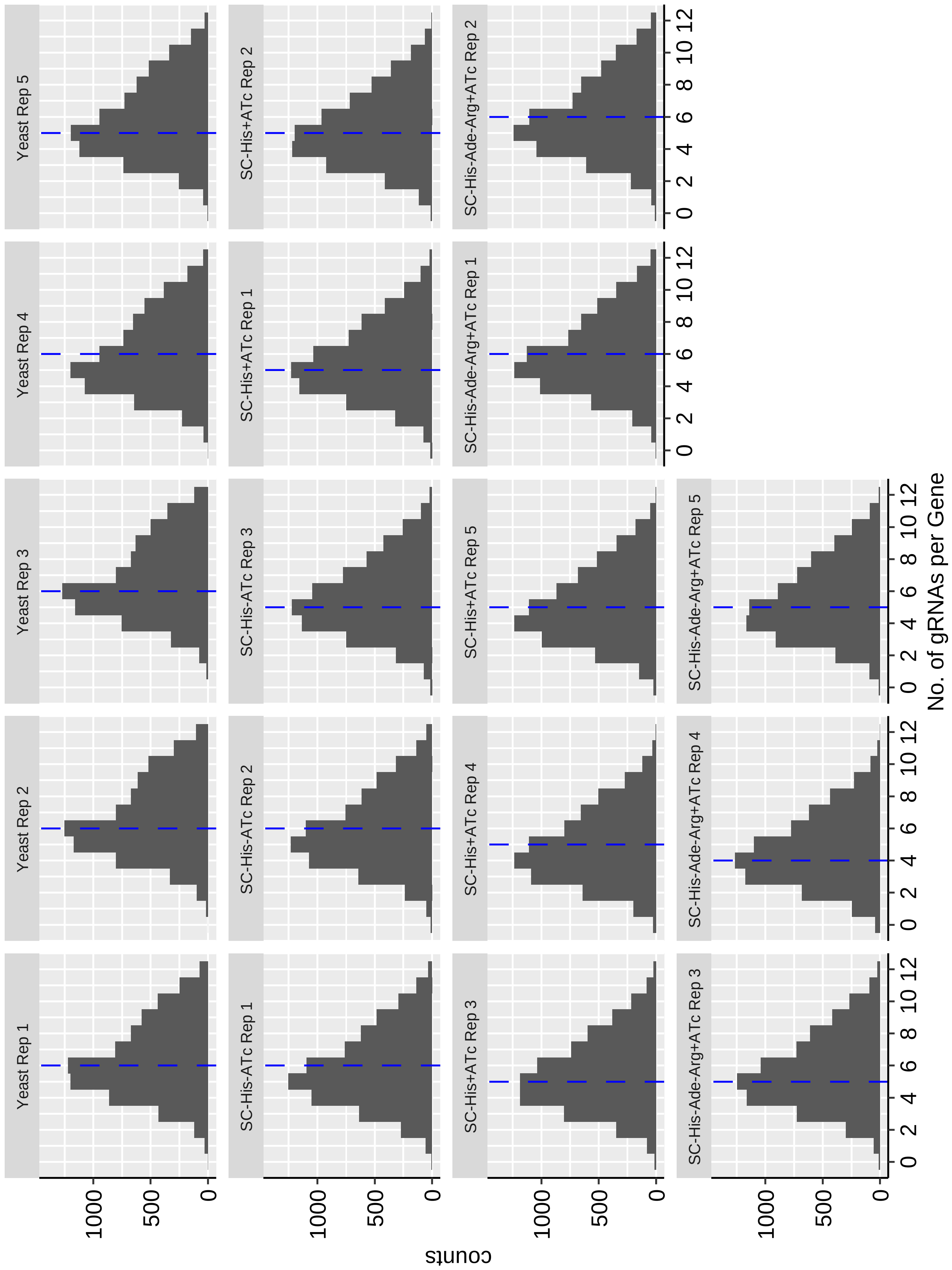

Supplementary Figure 4. Histogram depicting the number of gRNAs per gene in different samples. The dashed blue line denotes the median. Yeast Rep 1-5 are the yeast transformants.

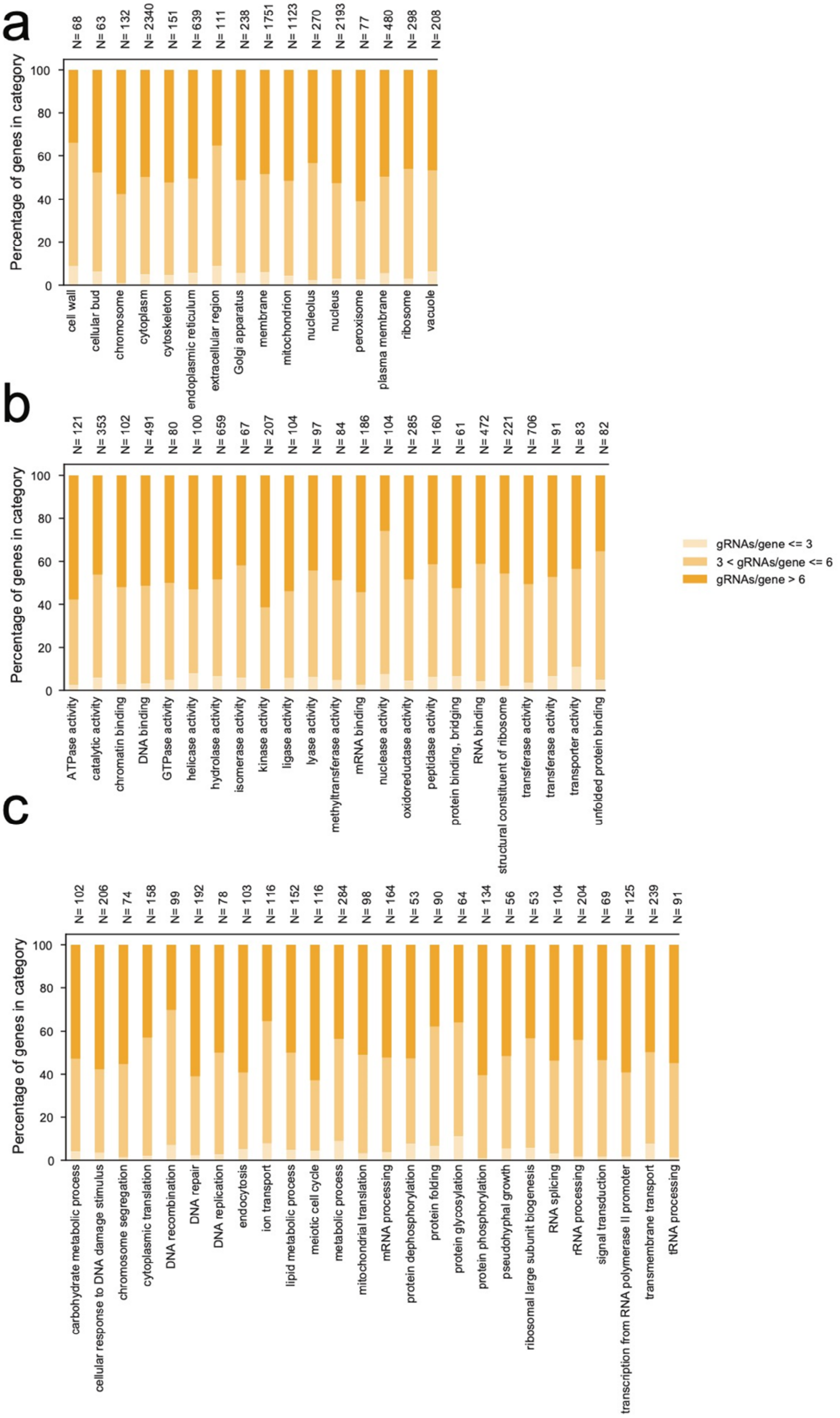

Supplementary Figure 5. Distribution of gRNAs across different GO terms. a) The distribution of gRNAs per gene across different compartments does not show any bias. **b)** The distribution of gRNAs per gene across different molecular functions shows a good representation. **c)** The distribution of gRNAs per gene across different biological processes does not show any bias.

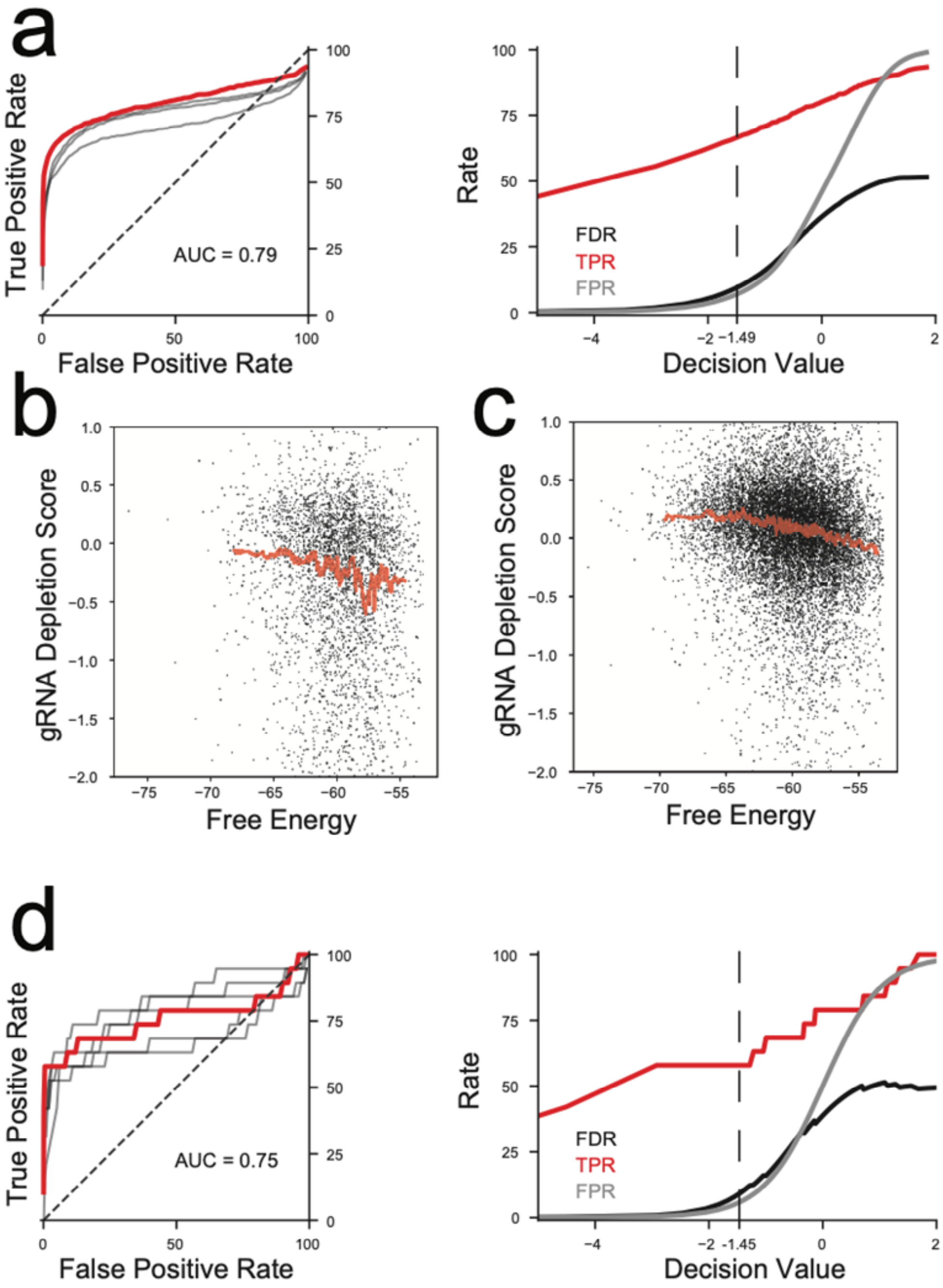

Supplementary Figure 6. Detection of dosage-sensitive genes, and the biosynthetic genes for adenine and arginine. a) ROC curve analysis for the detection of essential genes and FDR, TPR and FPR trends based on decision values. ROC curve shows that the depletion z score is a good classifier for essential genes. The individual replicates are shown in grey. Area under the curve is 0.79. Dashed line denotes the decision value for FDR<10%. **b)** Scatter plot depicting the gRNA depletion score for gRNAs targeting dosage sensitive essential genes versus the sgRNA secondary structure free energy. The red line represents the rolling average (window of 200). **c)** Scatter plot depicting the gRNA depletion score for gRNAs targeting nonessential genes versus the sgRNA secondary structure free energy. The red line represents the rolling average (window of 200). **d)** ROC curve for the detection of adenine and arginine biosynthesis genes and FDR, TPR and FPR trends based on decision values. ROC curve shows that the depletion z score is a good classifier for adenine and arginine biosynthetic genes. The individual replicates are shown in grey. Area under the curve is 0.75. Dashed line denotes the decision value for FDR<10%.

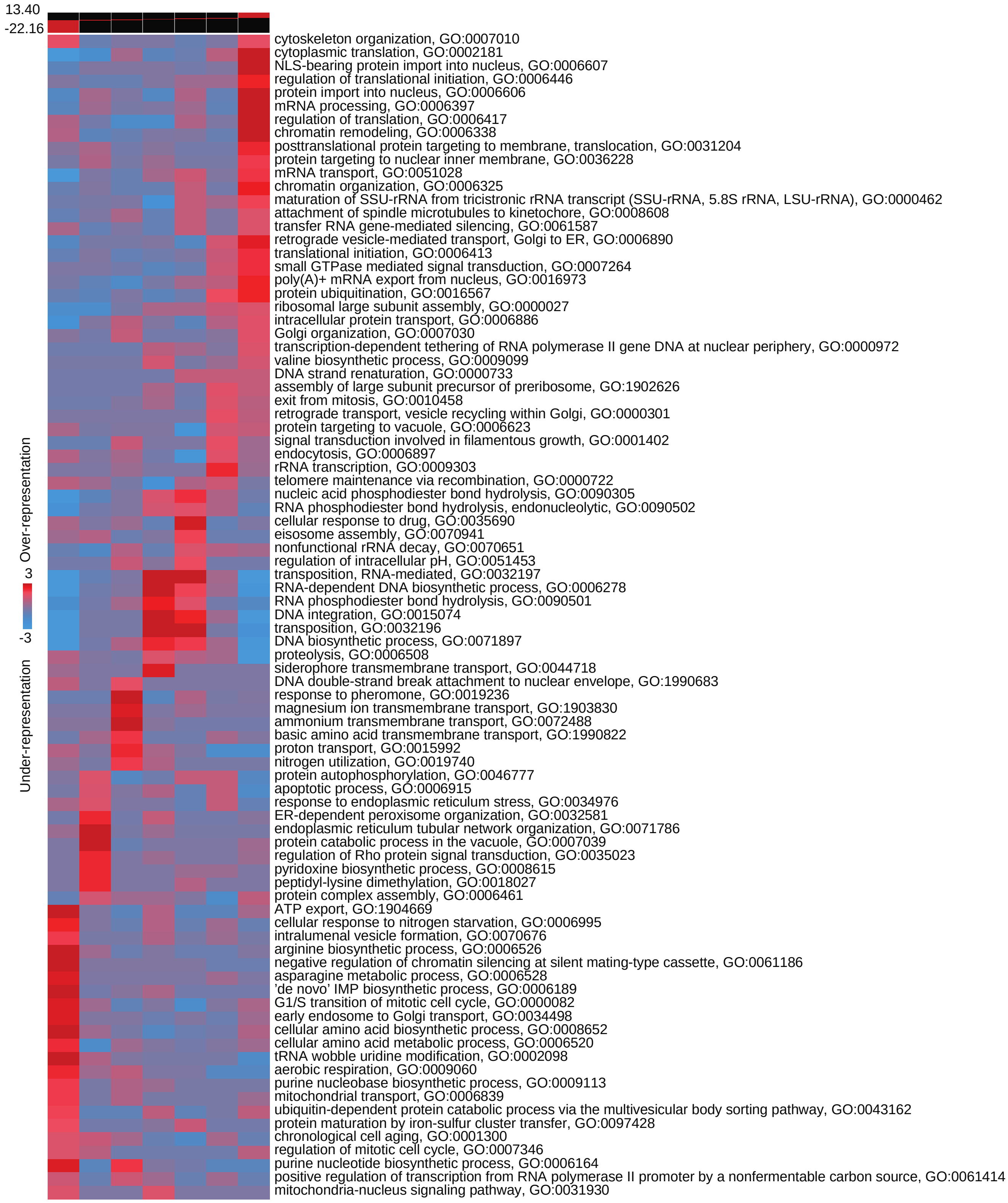

Supplementary Figure 7. iPAGE pathway analysis. iPAGE pathway analysis results shows all the biological processes that are perturbed in a statistically significant manner as a result of adenine and arginine starvation (p-value <0.05, random shuffling).
