# Supplementary material for: An inducible CRISPR interference library for genetic interrogation of Saccharomyces cerevisiae biology
Source: Commun Biol. 2020 Nov 27;3:723. doi: 10.1038/s42003-020-01452-9 (PMC7695836; doi:10.1038/s42003-020-01452-9)
Supplement: Supplementary file 3 — Description of Additional Supplementary Files [file 42003_2020_1452_MOESM3_ESM.pdf]

Description of Additional Supplementary Files

Name: **Supplementary Data 1**

Description: **Sheet (1)** Raw Data for figure 1b. **Sheet (2)** Raw Data for figure 1c.

Name: **Supplementary Data 2**

Description: Sequences of the library spacers.

Name: **Supplementary Data 3**

Description: Summary of the control synthetic randomly shuffled spacers.

Name: **Supplementary Data 4**

Description: Summary of frequency of reads in each sample.

Name: **Supplementary Data 5**

Description: **Sheet (1)** Gene depletion scores for samples with ATc versus without ATc. **Sheet (2)** Gene depletion scores for samples in the drop-out media for histidine, arginine and adenine versus histidine drop-out media. **Sheet (3)** Gene depletion scores for samples in the drop-out media for histidine, arginine and adenine versus histidine drop-out media for gRNAs with PAM between 0 and 150 bp upstream of TSS. **Sheet (4)** Non-essential genes that exhibit dosage-sensitivity. 76% of these genes were previously identified as having decreased fitness when mutated. For those genes the reference is cited.

Name: **Supplementary Data 6**

Description: List of the gene categories used in this study

Name: **Supplementary Data 7**

Description: List of the primer sequences used in this study
