# Supplementary material for: An inducible CRISPR interference library for genetic interrogation of Saccharomyces cerevisiae biology
Source: Commun Biol. 2020 Nov 27;3:723. doi: 10.1038/s42003-020-01452-9 (PMC7695836; doi:10.1038/s42003-020-01452-9)
Supplement: Supplementary file 11 — Reporting Summary [file 42003_2020_1452_MOESM11_ESM.pdf]

nature research

Corresponding author(s): Saeed Tavazoie

Last updated by author(s): Oct 18, 2020

Reporting Summary

Nature Research wishes to improve the reproducibility of the work that we publish. This form provides structure for consistency and transparency in reporting. For further information on Nature Research policies, see our Editorial Policies and the Editorial Policy Checklist.

Statistics

For all statistical analyses, confirm that the following items are present in the figure legend, table legend, main text, or Methods section.

n/a	Confirmed	
☐	☒	The exact sample size (*n*) for each experimental group/condition, given as a discrete number and unit of measurement
☐	☒	A statement on whether measurements were taken from distinct samples or whether the same sample was measured repeatedly
☐	☒	The statistical test(s) used AND whether they are one- or two-sided<br>*Only common tests should be described solely by name; describe more complex techniques in the Methods section.*
☐	☒	A description of all covariates tested
☐	☒	A description of any assumptions or corrections, such as tests of normality and adjustment for multiple comparisons
☐	☒	A full description of the statistical parameters including central tendency (e.g. means) or other basic estimates (e.g. regression coefficient) AND variation (e.g. standard deviation) or associated estimates of uncertainty (e.g. confidence intervals)
☐	☒	For null hypothesis testing, the test statistic (e.g. *F*, *t*, *r*) with confidence intervals, effect sizes, degrees of freedom and *P* value noted<br>*Give P values as exact values whenever suitable.*
☒	☐	For Bayesian analysis, information on the choice of priors and Markov chain Monte Carlo settings
☒	☐	For hierarchical and complex designs, identification of the appropriate level for tests and full reporting of outcomes
☐	☒	Estimates of effect sizes (e.g. Cohen's *d*, Pearson's *r*), indicating how they were calculated

Our web collection on statistics for biologists contains articles on many of the points above.

Software and code

Policy information about availability of computer code

Data collection	We used Illumina nextseq 500 series and the associated software to collect the data.
Data analysis	A custom computer code was used to analyze the data. The script was written in Python. In addition, we used cutadapt and bowtie2 to analyze the sequencing data. The random forest classifier was written in Python. The custom developed code to analyze the sequencing data is available on our GitHub at https://github.com/tavalab/yeast-CRISPRi.

For manuscripts utilizing custom algorithms or software that are central to the research but not yet described in published literature, software must be made available to editors and reviewers. We strongly encourage code deposition in a community repository (e.g. GitHub). See the Nature Research guidelines for submitting code & software for further information.

Data

Policy information about availability of data

All manuscripts must include a data availability statement. This statement should provide the following information, where applicable:

- Accession codes, unique identifiers, or web links for publicly available datasets
- A list of figures that have associated raw data
- A description of any restrictions on data availability

All the data is available in the supplementary tables. The raw sequencing data that support the findings are available at GEO accession: GSE159409.

Field-specific reporting

Please select the one below that is the best fit for your research. If you are not sure, read the appropriate sections before making your selection.

- [x] Life sciences
- [ ] Behavioural & social sciences
- [ ] Ecological, evolutionary & environmental sciences

For a reference copy of the document with all sections, see nature.com/documents/nr-reporting-summary-flat.pdf

Life sciences study design

All studies must disclose on these points even when the disclosure is negative.

Sample size	We have created two independently cloned library replicates. These replicates were transformed into five yeast replicates. The number of the yeast replicates, minimum of three, conforms with the current standards for CRISPRi library studies.
Data exclusions	For each sample, the read counts were converted to frequencies. The read frequencies below 10^-5 in both test and control samples were excluded to reduce noise. One of the replicates for RT-qPCR on Sec14 sample was excluded for being an outlier (Not within mean +- x standard deviation).
Replication	As presented in the text, we have created two independently cloned library replicates. These libraries were transformed into five yeast replicates. In each experiment we have a minimum of three replicates. The replicates were highly correlated. The correlations are shown in the manuscript.
Randomization	To create the synthetic scrambled genes, we utilized the gRNA depletion scores of the synthetic randomly shuffled gRNAs. We simulated a pool of synthetic scrambled genes by sampling the depletion scores of the synthetic randomly shuffled gRNAs, while maintaining the distribution of gRNA per gene in the sequenced CRISPRi library. Synthetic scrambled genes were populated by considering each yeast ORF and replacing its corresponding gRNAs depletion scores from the pool of synthetic randomly shuffled gRNAs. This process was repeated 200 times to generate a distribution of synthetic scrambled genes.
Blinding	Blinding was not relevant to our study as we did not perform any experiments needing blinding.

Reporting for specific materials, systems and methods

We require information from authors about some types of materials, experimental systems and methods used in many studies. Here, indicate whether each material, system or method listed is relevant to your study. If you are not sure if a list item applies to your research, read the appropriate section before selecting a response.

Materials & experimental systems

n/a	Involved in the study	
x		Antibodies
x		Eukaryotic cell lines
x		Palaeontology and archaeology
x		Animals and other organisms
x		Human research participants
x		Clinical data
x		Dual use research of concern

Methods

n/a	Involved in the study	
x		ChIP-seq
x		Flow cytometry
x		MRI-based neuroimaging
